# The role of neighbourhood greenspace quantity on mental health and cognitive development in early to middle childhood: a multilevel growth curve analysis of the UK Millennium Cohort Study

**DOI:** 10.1111/camh.12767

**Published:** 2025-03-24

**Authors:** Georgia Cronshaw, Emily Midouhas, Peninah Murage, Eirini Flouri

**Affiliations:** ^1^ Department of Psychology and Human Development IOE, UCL's Faculty of Education and Society London UK; ^2^ Public Health Environment and Society Department London School of Hygiene and Tropical Medicine London UK

**Keywords:** Longitudinal, child development, greenspace, mental health, cognitive ability

## Abstract

**Background:**

Childhood shapes lifelong wellbeing, making it crucial to understand how environmental factors impact development. This study examines the impact of neighbourhood greenspace quantity on the trajectories of emotional, behavioural and cognitive outcomes across childhood (at ages 3, 5, 7 and 11 years) with data from the UK's Millennium Cohort Study.

**Methods:**

Using multilevel growth curve models, we assessed the role of neighbourhood greenspace in small standard areas on trajectories of conduct problems, hyperactivity/inattention, peer problems, emotional symptoms and cognitive ability.

**Results:**

There was no direct association between greenspace and these child outcomes at the intercept (~ aged 7 years). However, greenspace was related to the slope of both conduct problems and cognitive ability, suggesting possible benefits in the early years, mainly before the start of formal education.

**Conclusion:**

The study highlights the potential effect of greenspace quantity on child development, but in the context of age. Longitudinal research tracking outcomes beyond childhood can shed more light on age‐related effects of greenspace across areas of development.

**Key Practitioner Message**

**What is known?**

- Over the last two decades, there has been a growing interest in the impact of environmental exposures, including neighbourhood greenspace, on children's mental health and cognitive development.
- While there is strong evidence of greenspace's positive effects, the specific ways it influences children's mental health and cognitive abilities are still not fully understood.

**What is new?**

- Less attention has been given to the dynamic nature of children's exposure to greenspace. It is important to consider how a child's exposure to greenspace changes over time (e.g. moving house).
- This study used data from the UK Millennium Cohort Study to examine how neighbourhood greenspace affects child development in a changing environment, accounting for residential mobility and assessing how shifts in greenspace exposure influence developmental outcomes over time.

**What is significant for clinical practice?**

- Understanding the dynamic relationship between greenspace exposure and child development has implications for clinical practice. Clinicians can use this knowledge to encourage families, particularly those with young children, to spend more time in green spaces, especially during the early primary school years.

## Background

Early childhood is a critical period for development, where environmental factors significantly shape cognitive, emotional and behavioural growth (Lämmle, Woll, Mensink, & Bös, 2013; Richter et al., 2019). Greenspace, like parks and woodlands, is one such influential environmental factor (Barton & Rogerson, 2017). Access to green spaces has been associated with improved physical, mental and cognitive outcomes in adults (Public Health England, 2020). These benefits are attributed to both direct mechanisms (e.g. stress reduction, attention restoration; Kaplan, 1995; Ulrich, 1984) and indirect factors (e.g. promoting physical activity, reducing pollution, enhancing social interaction; Calderón‐Garcidueñas et al., 2011; Fjørtoft, 2001; Freire et al., 2010; Hartig, 2021; Markevych et al., 2017).

However, evidence on the impact of greenspace exposure on child development is mixed (e.g. Andrusaityte, Grazuleviciene, Dedele, & Balseviciene, 2020; Madzia et al., 2019; Markevych et al., 2014; Naya, Yi, Chu, Dunton, & Mason, 2022). Several cross‐sectional studies suggest positive associations between greenspace and emotional and behavioural outcomes. For instance, Amoly et al. (2014) linked residential greenness to reduced ADHD symptoms, while Zach et al. (2016) observed reduced mental health risks in children with greenspace access. However, not all studies have shown consistent benefits. Nordbø, Raanaas, Nordh, and Aamodt (2020) found that recreational greenspace was associated with higher depressive symptoms in 8‐year‐olds, though these effects were partly mitigated by physical and social activities. Similarly, Mueller and Flouri (2021) reported no direct link between greenspace and mental health outcomes, though they identified a relationship between higher greenness and lower self‐esteem in children without gardens. Longitudinal studies further complicate the picture, with some studies finding minimal or no associations between greenspace and emotional or behavioural development (Ahmed et al., 2022; Flouri, Midouhas, & Joshi, 2014; Madzia et al., 2019; Richardson, Pearce, Shortt, & Mitchell, 2017).

In terms of cognitive outcomes, greenspace exposure is often linked to improvements in cognitive ability, such as enhanced spatial working memory (Flouri, Papachristou, & Midouhas, 2019) and attention (Saenen et al., 2023). However, not all studies confirm these findings. For example, cross‐sectionally, Buczyłowska, Baumbach, et al. (2023) found no consistent links between greenspace exposure and intelligence in children aged 10–13, even after considering ADHD status. Similarly, longitudinal studies report mixed results; Francesconi, Flouri, and Kirkbride (2022) found no significant associations between greenspace and cognitive ability. Conversely, others suggest potential benefits, such as reduced inattentiveness (Dadvand et al., 2015). However, Anabitarte et al. (2022) observed a cross‐sectional association between residential greenspace and reduced inattentiveness, yet no longitudinal associations with attentional function were found.

These inconsistencies have been documented in several systematic reviews, which synthesise the mixed findings (Buczyłowska, Zhao, et al., 2023; Islam, Johnston, & Sly, 2020; Luque‐García, Corrales, Lertxundi, Díaz, & Ibarluzea, 2022; McCormick, 2017; Vanaken & Danckaerts, 2018; Zare Sakhvidi et al., 2022). These discrepancies may stem from variations in how greenspace is measured. Most studies assess greenspace based on the amount or proximity of greenery around a family's residence, which may oversimplify the complex, multidimensional nature of greenspace exposure. Moreover, many studies rely on cross‐sectional designs, which fail to capture the dynamic and temporal aspects of child development (Sprague, Bancalari, Karim, & Siddiq, 2022). Longitudinal studies, though fewer in number, have also yielded inconsistent results (e.g. Ahmed et al., 2022; Feng & Astell‐Burt, 2017; Flouri et al., 2014; Richardson et al., 2017), with discrepancies in the timing and duration of greenspace exposure. The impact of greenspace may vary significantly depending on when and for how long children are exposed, particularly as they transition through different life stages (e.g. starting school, moving homes).

### The present study

This study uses data from the UK's Millennium Cohort Study (MCS) and growth curve modelling to explore how neighbourhood greenspace impacts children's cognitive ability and mental health from ages 3 to 11 years. We hypothesise that more greenspace will be associated with better outcomes at age 7 (the ‘intercept’), with the association potentially changing as children age (the ‘slope’). We therefore also examine whether this relationship varies over time, captured by the ‘greenspace*age’ interaction term.

## Methods

### Sample

MCS is a national longitudinal birth‐cohort study that tracks ~ 19,000 children born in England, Scotland, Wales and Northern Ireland from 2000 to 2002. The design oversampled specific groups, ensuring representation from families outside England, disadvantaged areas, and ethnic minority groups. MCS provided weights, stratifying, and clustering variables to address attrition and complex sampling (Plewis, 2007).

Eight survey sweeps were conducted, starting when children were about 9 months old, with follow‐ups at ages 3, 5, 7, 11, 14, 17, and 23 years. Ethical approval was granted by the NHS Research Ethics Committee and the UCL Institute of Education.

This study used data from sweeps two to five, focusing on singletons and firstborns of twins/triplets, using the main respondent's answers for parent‐reported measures. The analytic sample consisted of 6946 cohort members who continuously resided in England (S2–25), avoiding between‐country geographic confounding (see Figure S1).

### Measures

#### Outcomes

##### Child emotional and behavioural difficulties

Child emotional and behavioural difficulties were assessed at ages 3, 5, 7 and 11 using the parent‐reported Strengths and Difficulties Questionnaire (SDQ; Goodman, 1997). The SDQ is a reliable instrument widely recognised for its capacity to measure both emotional and behavioural problems in children (Mieloo et al., 2012). The SDQ comprises 25 items that assess emotional and behavioural difficulties, employing a 3‐point scale ranging from 0 (not true) to 2 (certainly true). It encompasses five domains: (1) Emotional Symptoms; (2) Conduct Problems; (3) Hyperactivity/Inattention; (4) Peer Problems; and (5) Prosocial Behaviour. In this study, subscales one through four (indexing emotional and behavioural difficulties) were utilised. For ages 3, 5, 7 and 11, the internal consistency (Cronbach's alpha) of the measures ranged from 0.56–0.68 for conduct problems, 0.71–0.79 for hyperactivity/inattention, 0.47–0.64 for peer problems and 0.52–0.71 for emotional symptoms. While most of these values are considered adequate and have been previously reported in this cohort (Flouri et al., 2014), due to concerns about internal consistency, we refrained from drawing conclusions on peer problems.

##### Child cognitive ability

Child cognitive ability was assessed at ages 3, 5, 7 and 11 using cognitive measures to evaluate general cognitive development. At age 3 (Sweep 2), the assessments included the British Ability Scales (BAS) Naming Vocabulary, which measures expressive language ability, and the Bracken School Readiness Assessment, targeting foundational concepts prior to formal schooling. At age 5 (Sweep 3), the BAS Naming Vocabulary was used again, along with BAS Picture Similarities, measuring non‐verbal reasoning, and BAS Pattern Construction, which assesses spatial problem‐solving, spatial awareness, and hand‐eye coordination. At age 7 (Sweep 4), BAS Pattern Construction was used once more, in addition to BAS Word Reading, which measures English reading ability, and the National Foundation for Educational Research (NFER) number skills assessment to determine mathematical ability. By age 11 (Sweep 5), cognitive ability was assessed using BAS Verbal Similarities, measuring verbal reasoning and knowledge, and the CANTAB spatial working memory test. PCA was used to construct a cognitive ability variable from the cognitive measures at each sweep. Components were selected based on eigenvalues greater than 1, along with total variance explained and scree plot results. This method is consistent with prior MCS studies/reports (Connelly, 2013; Francesconi et al., 2022). Each component score was then standardised to a mean of 100 and a standard deviation of 15.

#### Exposure

##### Neighbourhood greenspace

Neighbourhood (ward) greenspace data were obtained from the Multiple Environmental Deprivation Index (MEDIx; http://cresh.org.uk/cresh‐themes/environmental‐deprivation/medix‐and‐medclass/). Wards are a block of UK electoral geography and typically have around 5500 residents (http://www.ons.gov.uk/ons/guide‐method/geography/beginner‐s‐guide/administrative/england/electoral‐wards‐divisions/index.html). For each UK ward, MEDIx calculated the quantity of greenspace via the combination of data from the Generalised Land Use Database (GLUD) and the Coordination of Information on the Environment (CORINE) (Richardson, Mitchell, Shortt, Pearce, & Dawson, 2010; https://cresh.org.uk/cresh‐themes/green‐spaces‐and‐health/ward‐level‐green‐space‐estimates/). GLUD categorises land use in England into nine categories: greenspace, domestic gardens, fresh water, domestic buildings, non‐domestic buildings, roads, paths, railways, and other. It includes all vegetated areas larger than 5 m^2^ (excluding private gardens), regardless of accessibility. CORINE is a UK land cover dataset from 2000, derived from remotely sensed satellite imagery, sensitive only to larger green spaces (e.g. parks) as it does not capture areas smaller than roughly 1 ha. The MEDIx ward‐level greenspace data are available in deciles from 1 to 10, where 10 represents areas with the most greenspace and 1 represents those with the least. This data, already linked to the MCS, integrates neighbourhood‐level environmental data with individual‐level participant data, as used in previous studies (Flouri et al., 2014; Francesconi et al., 2022; Mueller & Flouri, 2021).

#### Confounders

Unless specified, confounders were derived from the main respondent at baseline (age 3) and selected based on their relevance to both greenspace exposure and outcomes, which primarily reflect socioeconomic factors (Badini et al., 2024; Generaal, Timmermans, Dekkers, Smit, & Penninx, 2019; Zhang et al., 2024). The *family‐level* variables included: maternal education (Mother university educated or not); family structure (Lives with both natural parents or not); home ownership (family owns a home outright/mortgage or not); poverty indicator (living below or above 60% of median income); sole access to a garden (sole access to a garden or not) at each sweep; residential mobility (moved house or not) at each sweep, and maternal psychological distress, measured with the Kessler 6 Psychological Distress Scale (Kessler et al., 2002). The *neighbourhood‐level* variables (assessed at each sweep) were MEDIx ward‐level air pollution (PM10), given in deciles, and a urbanicity/rurality variable (living in an urban area or not), using the Office for National Statistics 2005 area classification data for England. Finally, the *child‐level* variables included sex (male and female), age in years, and ethnicity (White, Black, Indian, Pakistani/Bangladeshi, Mixed, and Other). Finally, the MCS stratification variable was used to account for the complex survey design, given as England Advantaged, England Disadvantaged and England Ethnic.

### Statistical analysis

First, a bias analysis was conducted to determine whether there were differences between the analytic and non‐analytic samples. The aim of the bias analysis was to examine the degree to which the analytic sample was representative of MCS. We then went on to consider the missingness within the analytic sample. This was followed by a correlation analysis between exposure and outcomes within the analytic sample, and finally, growth curve modelling. Our models were three‐level, with outcomes (level 1), nested in children (level 2), nested in neighbourhoods (wards) (level 3). Models required at least one measure of the outcome variable across each sweep. We fitted both fixed and random linear slopes. In addition to a linear age term, we included a quadratic age term in the fixed part of the models for the four SDQ outcomes to account for the curved shapes of children's average trajectories of these emotional and behavioural problems across our study period. By specifying a random linear slope on the child's age, allowing for changes in child outcomes across time to vary between children, we were able to model individual trajectories of child outcomes from ages 3–11. In all these analyses, the models were sequentially adjusted, moving from the unconditional to the fully adjusted model (Table S1). In all models, age was centred at the grand mean (age 6.672 years, ~ age 7) to enhance interpretability. Missing values (ranging from 0% to 15.6% in the analytic sample) were imputed using chained equations and twenty imputed datasets were produced. In the imputation model, we used all covariates as both predictor and predicted variables, as well as MCS design variables as predictor variables. We then analysed the imputed datasets in Stata/MP 18.0, using the *mi estimate* command. This command performs separate analyses on each imputed dataset, then aggregates the estimates and their Variance–Covariance matrices, applying Rubin's rules (1996) to produce final coefficient estimates.

## Results

### Descriptive statistics

Our bias analysis indicated potential bias (Tables 1 and 2). Specifically, compared with those excluded, the families included in the analytic sample lived in less green and more polluted areas. Additionally, the children in the analytic sample showed higher cognitive ability and predominantly lower hyperactivity/inattention, conduct, and peer problems, although they had comparable levels of emotional symptoms. The correlations between greenspace and child outcomes in the analytic sample exhibited the expected trends, although they were modest in size (Tables S2 and S3). Specifically, at all measured time points (ages 3, 5, 7 and 11 years), children in greener areas had fewer emotional, conduct, hyperactivity/inattention and peer problems and higher cognitive ability.

**Table 1:** Descriptive and bias analysis (categorical variables)

Characteristics	Analytic sample (*n* = 6946)		Non‐analytic sample (*n* = 12,298)		Chi‐squared
	*n*	%	*n*	%	*F* ^a^
Male	3465	49.88	6430	52.28	8.08**
Ethnicity					
White	5426	78.12	10,316	84.07	7.40**
Mixed	248	3.57	341	2.78	1.11
Indian	268	3.86	229	1.87	5.03*
Pakistani/Bangladeshi	609	8.77	741	6.04	1.49
Black	262	3.77	468	3.81	10.78**
Other	133	1.91	175	1.43	1.68
Below the poverty line	2068	30.08	3012	35.40	79.82***
Mother university educated	1305	18.86	1676	13.73	64.29***
Family owns home (mortgage/outright)	4677	67.82	5169	60.58	98.42***
Lives with both natural parents	5777	83.58	6575	76.83	104.69***
Sole access to garden (S2)	6275	90.60	10,384	84.90	185.54***
Sole access to garden (S3)	6324	91.28	10,541	86.15	186.08***
Sole access to garden (S4)	6352	91.67	10,580	86.47	167.13***
Sole access to garden (S5)	6409	92.47	10,725	87.63	169.71***
Moved House (S2; baseline)	–	–	–	–	–
Moved house (S3)	921	13.27	1271	15.88	44.81***
Moved house (S4)	607	8.74	754	10.93	28.05***
Moved house (S5)	1245	17.93	1560	24.63	138.60***

*N* and % are unweighted, with percentages based on complete cases for each sample. MCS is a UK‐wide cohort study. The analytic sample (England) uses different urbanicity/rurality measures than the rest of the UK, so direct comparisons between the analytic and non‐analytic samples are not possible.

^a^ *F* statistic for design‐based Chi‐square was converted to an *F* test to account for the MCS sampling design.

*p < .05; **p < .01; ***p < .001.

**Table 2:** Descriptive and bias analysis (continuous variables)

Characteristics	Analytic sample (*n* = 6946)		Non‐analytic sample (*n* = 12,298)		*T* ‐test
	Mean	*SD*	Mean	*SD*	*t* ^a^
Greenspace (Ward Level)					
Greenspace (S2)	4.48	2.17	4.73	3.21	−2.20*
Greenspace (S3)	4.57	2.19	4.82	3.29	−2.13*
Greenspace (S4)	4.64	2.20	4.95	3.47	−2.44*
Greenspace (S5)	4.72	2.20	5.03	3.58	−2.27*
Age (years)					
Age (S2)	3.11	0.15	3.16	0.26	−10.96***
Age (S3)	5.19	0.20	5.23	0.31	−5.00***
Age (S4)	7.22	0.30	7.24	0.21	−2.85*
Age (S5)	11.15	0.29	11.19	0.42	−4.96***
Emotional symptoms					
Emotional symptoms (S2)	1.29	1.20	1.36	1.79	−2.43*
Emotional symptoms (S3)	1.32	1.32	1.38	1.91	−1.82
Emotional symptoms (S4)	1.48	1.48	1.49	2.19	−0.07
Emotional symptoms (S5)	1.83	1.70	1.83	2.55	−0.05
Peer problems					
Peer problems (S2)	1.45	1.33	1.53	1.85	−2.54*
Peer problems (S3)	1.03	1.17	1.19	1.78	−4.36***
Peer problems (S4)	1.15	1.32	1.21	1.54	−1.46
Peer problems (S5)	1.28	1.44	1.39	2.21	−2.46*
Conduct problems					
Conduct problems (S2)	2.70	1.72	2.82	2.46	−2.96**
Conduct problems (S3)	1.40	1.24	1.56	1.85	−5.51***
Conduct problems (S4)	1.29	1.25	1.45	1.99	−4.52***
Conduct problems (S5)	1.30	1.31	1.43	2.11	−3.61***
Hyperactivity/inattention					
Hyperactivity/inattention (S2)	3.81	1.99	3.94	2.85	−2.83**
Hyperactivity/inattention (S3)	3.15	1.98	3.37	2.90	−4.34***
Hyperactivity/inattention (S4)	3.22	2.13	3.46	3.19	−3.96***
Hyperactivity/inattention (S5)	3.00	2.07	3.18	3.22	−3.09**
Cognitive ability					
Cognitive ability (S2)	101.02	15.13	99.18	14.85	7.09***
Cognitive ability (S3)	101.05	14.11	99.10	15.65	8.06***
Cognitive ability (S4)	101.23	14.86	98.71	15.04	9.72***
Cognitive ability (S5)	100.87	14.99	99.03	14.95	6.86***
Maternal psychological distress (Kessler 6)	3.09	2.91	3.22	4.52	−1.67
Air pollution (S2)	7.26	1.97	5.77	3.69	14.17***
Air pollution (S3)	7.20	1.96	5.59	3.74	15.02***
Air pollution (S4)	7.16	1.96	5.23	3.88	16.16***
Air pollution (S5)	7.11	1.98	5.06	3.99	15.53***

Means are weighted to account for the MCS sampling design. *SD*, standard deviation.

^a^ *T* test statistic.

*p < .05; **p < .01; ***p < .001.

### Growth curve models

The objective of our study was to examine the impact of neighbourhood greenspace quantity on child outcomes and how this relationship changes over time. The unconditional model is shown in Table S4. In the minimally adjusted model (model 1; Table S5), results for peer problems and hyperactivity/inattention were not significant for either the association of greenspace at the intercept or for the greenspace*age interaction term (slope). A significant association at the intercept was seen in the minimally adjusted model for emotional symptoms (*b* = −0.018, *SE* = 0.007, *p* < .05). As greenspace increased, emotional symptoms decreased, but no significant greenspace*age interaction was found. This relationship was fully attenuated after adjusting for neighbourhood characteristics and garden access (model 2; Table S6). Stepwise adjustment revealed that the time‐varying urbanicity/rurality variable fully explained the association.

Although the greenspace–intercept association was not significant, the greenspace*age (slope) interaction was significant for both conduct problems and cognitive ability (model 1; Table S5). After full adjustment (Table 3), a positive greenspace*age effect persisted for conduct problems (*b* = 0.003, *SE* = 0.001, *p* < .05), and a negative effect persisted for cognitive ability (*b* = −0.099, *SE* = 0.020, *p* < .001), confirming the robustness of these effects.

**Table 3:** Fixed and random effects estimates from models predicting emotional symptoms, peer problems, conduct problems, hyperactivity/inattention and cognitive ability (*n* = 6946)

Model 4 (fully adjusted model)	Emotional symptoms		Peer problems		Conduct problems		Hyperactivity/inattention		Cognitive ability	
	*b* (*SE*)	*p*	*b* (*SE*)	*p*	*b* (*SE*)	*p*	*b* (*SE*)	*p*	*b* (*SE*)	*p*
Fixed effects										
Age	0.062*** (0.009)	.000	−0.031*** (0.007)	.000	−0.228*** (0.007)	.000	−0.116*** (0.010)	.000	0.477*** (0.107)	.000
Age^2^	0.006*** (0.001)	.000	0.018*** (0.001)	.000	0.053*** (0.001)	.000	0.017*** (0.002)	.000	–	–
Greenspace	0.002 (0.009)	.854	0.011 (0.009)	.204	0.003 (0.010)	.748	0.015 (0.012)	.241	−0.083 (0.089)	.348
Greenspace × Age	0.000 (0.002)	.975	0.000 (0.001)	.951	0.003* (0.001)	.015	0.002 (0.002)	.267	−0.099*** (0.020)	.000
England Disadvantaged (ref. England Advantaged)	0.126** (0.037)	.001	0.175*** (0.039)	.000	0.213*** (0.044)	.000	0.136* (0.058)	.020	−1.691*** (0.481)	.000
England Ethnic (ref. England Advantaged)	0.052 (0.092)	.575	0.070 (0.070)	.316	0.041 (0.090)	.647	0.016 (0.140)	.908	−1.837* (0.850)	.031
Living in an Urban Area	0.103 (0.060)	.087	0.046 (0.058)	.422	0.111 (0.058)	.054	0.110 (0.090)	.220	−0.521 (0.559)	.351
Air Pollution (PM10)	0.000 (0.009)	.957	0.008 (0.009)	.381	−0.001 (0.012)	.923	0.009 (0.013)	.491	−0.022 (0.086)	.801
Sole Access to Garden	−0.064 (0.105)	.541	−0.106 (0.055)	.054	−0.092 (0.101)	.364	−0.177 (0.123)	.151	0.089 (0.537)	.868
Female	0.032 (0.031)	.306	−0.238*** (0.028)	.000	−0.331*** (0.032)	.000	0.749*** (0.051)	.000	1.938*** (0.272)	.000
Ethnicity Mixed (ref. Ethnicity White)	0.031 (0.100)	.756	0.035 (0.087)	.689	−0.140 (0.085)	.098	−0.194 (0.152)	.201	0.130 (0.975)	.894
Ethnicity Indian (ref. Ethnicity White)	0.079 (0.117)	.502	0.448*** (0.104)	.000	−0.105 (0.110)	.339	−0.024 (0.218)	.912	−2.532* (1.212)	.037
Ethnicity Pakistani/Bangladeshi (ref. Ethnicity White)	0.479*** (0.122)	.000	0.612*** (0.078)	.000	−0.157 (0.081)	.053	0.170 (0.129)	.188	−8.196*** (0.854)	.000
Ethnicity Black (ref. Ethnicity White)	−0.267* (0.119)	.025	0.037 (0.128)	.770	−0.491** (0.148)	.001	−0.689*** (0.187)	.000	−4.275** (1.244)	.001
Ethnicity Other (ref. Ethnicity White)	0.009 (0.139)	.947	0.270 (0.180)	.133	−0.501*** (0.116)	.000	−0.301 (0.193)	.119	−2.215* (1.089)	.042
Moved House	0.080* (0.038)	.035	−0.006 (0.033)	.858	−0.028 (0.029)	.336	−0.012 (0.042)	.781	−0.180 (0.294)	.540
Below the poverty line	0.202*** (0.053)	.000	0.163** (0.052)	.002	0.196*** (0.054)	.000	0.192* (0.079)	.015	−3.429*** (0.422)	.000
Lives with both natural parents	−0.084 (0.058)	.147	−0.096 (0.056)	.085	−0.241*** (0.068)	.000	−0.355*** (0.098)	.000	0.661 (0.490)	.177
Owns Home	−0.198*** (0.045)	.000	−0.301*** (0.047)	.000	−0.389*** (0.049)	.000	−0.428*** (0.069)	.000	3.374***(0.416)	.000
Mother University Educated	−0.126** (0.040)	.002	−0.182*** (0.036)	.000	−0.301*** (0.047)	.000	−0.696*** (0.071)	.000	5.995*** (0.383)	.000
Maternal Psychological Distress	0.083*** (0.006)	.000	0.068*** (0.005)	.000	0.080*** (0.005)	.000	0.099*** (0.008)	.000	−0.141** (0.046)	.002
Constant	1.258*** (0.148)	.000	1.166*** (0.141)	.000	1.607*** (0.194)	.000	3.874*** (0.206)	.000	99.872*** (1.296)	.000
Random effects										
Level 2 (child)										
Between‐child intercept variance	1.106 (0.039)		0.878 (0.037)		1.128 (0.039)		2.820 (0.078)		81.744 (2.907)	
Between‐child slope variance	0.029 (0.002)		0.019 (0.002)		0.019 (0.001)		0.035 (0.002)		1.279 (0.102)	
Between‐child intercept/slope variance covariance	0.090 (0.007)		0.035 (0.005)		−0.041 (0.006)		0.039 (0.009)		0.954 (0.347)	
Level 3 (area)										
Variance	0.006 (0.005)		0.019 (0.007)		0.020 (0.011)		0.039 (0.013)		4.284 (0.717)	
Residual variance (level 1)	1.476 (0.045)		1.262 (0.036)		1.284 (0.031)		2.151 (0.044)		90.098 (2.057)	

*b*, unstandardised coefficients; *SE*, standard error.

*p < .05; **p < .01; ***p < .001.

**Figure 1:**
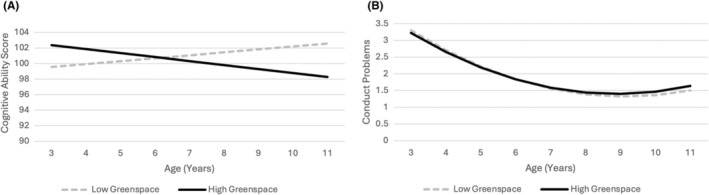
Predicted trajectories of cognitive ability scores (A) and conduct problems (B) by greenspace decile from ages 3–11 years The grey dashed line shows predicted scores in the lowest decile of greenspace, and the black solid line shows predicted scores in the highest decile of greenspace. Reference groups are used for categorical variables and means are used for continuous variables

To illustrate the relationships between greenspace, conduct problems and cognitive ability, predicted trajectories were plotted using the fixed effects of the growth curve models. As shown in Figure 1A, a child in the greenest area starts with higher cognitive ability, but scores converge in early primary school and then diverge, with the greenest area showing a decline and the least green area showing an increase. By age 11, the child in the greenest area has the lowest score, and the child in the least green area has the highest. Regarding conduct problems (Figure 1B), a child in a low greenspace area starts with slightly more issues, but both groups show a downward trend until early primary school, after which the child in the high greenspace area experiences a faster increase, leading to slightly higher conduct problems by age 11.

### Supplementary analysis

To explain these findings, we conducted five supplementary analyses. First, we compared results from imputed data and complete case analysis to assess sensitivity to missing values, finding the results robust (Table S8). We then explored whether the cognitive advantage and increased conduct problems in less green areas, or the advantage in greener areas during early years, were due to the ‘London effect’ (Gamsu, 2018), school differences (Baró, Camacho, Del Pérez Pulgar, Triguero‐Mas, & Anguelovski, 2021), rurality/urbanicity masking geographic variations and sex differences. Four additional models were therefore fitted for cognitive ability and conduct problems by adding: (1) time‐varying London resident status (i.e. lives in London or not); (2) time‐varying fee‐paying school status (i.e. school is fee‐paying or not); (3) time‐varying settlement type (‘urban > 10k ‐ less sparse’; ‘urban > 10k ‐ sparse’; ‘town and fringe – less sparse’; ‘town and fringe – sparse’; ‘village, hamlet and isolated dwellings – less sparse’; ‘village, hamlet and isolated dwellings ‐ sparse’), replacing time‐varying urbanicity/rurality, and (4) sex and greenspace interaction terms on the intercept and slope.

Despite additional analyses, the primary relationships for cognitive ability remained unchanged, with no supplementary analyses explaining the observed patterns (Tables S9–S12). For conduct problems, adding school type revealed greater differences, with a child in a high greenspace area showing fewer conduct problems. This difference persisted until age 10, unlike the main analysis, where convergence occurred at age 6 (Figure S2).

## Discussion

Our study examined the impact of neighbourhood greenspace on mental and cognitive outcomes among UK children aged 3–11. Contrary to expectations and prior research, we found no significant main effect of the quantity of greenspace on child outcomes. However, as anticipated, greenspace was associated with changes over time, particularly for conduct problems and cognitive ability, highlighting age‐related associations, which are consistent with other studies (Vanaken & Danckaerts, 2018).

The absence of a main effect is unexpected but aligns with findings by Mueller, Flouri, and Kokosi (2019) and Richardson et al. (2017). This may be due to two factors: First, the measure of greenspace used (ward‐level data excluding domestic gardens) may not capture the nuances of exposure. Factors, such as accessibility, proximity, use, and quality of greenspace could be more influential (Alderton et al., 2022) and warrant further exploration. Second, the relationship between greenspace and child development is likely complex, with moderating factors playing a role. For example, Flouri et al. (2014) found that greenspace was associated with fewer emotional problems in disadvantaged children aged 3–5. Similarly, Mueller and Flouri (2021) noted that greenspace affected mental health at age 11, but only under certain conditions (e.g. area safety, physical activity or garden access). This suggests that greenspace's impact on children's mental and cognitive development may be contingent on moderating factors.

Age was a key moderator in our study. While greenspace showed modest benefits for both cognitive ability and conduct problems in early childhood, these effects diminished around the start of primary school. By the later primary years, children in areas with less greenspace showed higher cognitive ability and fewer conduct problems. Previous research suggests that the relationship between greenspace and child outcomes may evolve with age (Balseviciene et al., 2014; Feng & Astell‐Burt, 2017; Mueller, Midouhas, & Flouri, 2023), possibly reflecting changing patterns of engagement with greenspace (Vanaken & Danckaerts, 2018). In early childhood, green spaces may support cognitive development and reduce conduct problems through active play or physical activity. However, as children enter formal education, academic activities likely become more central to cognitive development (Brod, Bunge, & Shing, 2017), possibly diminishing the cognitive benefits of greenspace. At the same time, as children age, their use of green spaces may shift from play to more passive or social activities (Ward Thompson, 2007), which may be less beneficial for cognition and could worsen behavioural outcomes. These changes may explain the observed decline in cognitive benefits and the modest rise in conduct problems. However, it is important to note that despite being significant, this slope effect was very small, suggesting that real‐world impacts may be very subtle.

Given the unexpected negative effects of high greenspace on cognitive ability and conduct problems in later childhood, we explored several potential confounding and moderating factors. Previous studies have also reported negative associations between greenspace and cognitive ability (Almeida, Barros, & Ribeiro, 2022; Beere & Kingham, 2017), often attributing them to unmeasured confounding (Browning, Kuo, Sachdeva, Lee, & Westphal, 2018). Thus, we examined three possibilities of confounding: “the London effect”, school type (fee‐paying vs. non‐fee‐paying), and settlement type, but these factors did not alter the results for cognitive ability. An additional analysis of sex as a moderator did not change the patterns for cognitive ability either, though it revealed a sex difference. Therefore, further research is needed to identify other moderating and confounding factors that might help explain the relationship observed. For conduct problems, a clearer positive effect emerged when school type was included as a confounder. Specifically, trajectories showed that in greener areas, conduct problems were fewer by the end, rather than the beginning, of primary school. This suggests that after controlling for school type, thus providing another layer of adjustment for family SES and likely own school‐level exposure to greenspace, children in greener areas showed fewer conduct problems – though the difference was subtle.

Our study has several limitations. First, as an observational study, it cannot establish causality. Second, our cognitive measures may have been too broad, and more targeted studies using specific cognitive domains could offer deeper insights (Buczyłowska, Zhao, et al., 2023). Third, parent‐reported mental health measures may not fully capture children's emotional and behavioural issues, especially outside the home environment. Fourth, although our large sample improves generalisability, it was skewed towards wealthier families with more educated mothers, which should be considered when making broad conclusions. Finally, our measure of greenspace focused solely on quantity, not considering other factors like type, quality, or accessibility, which might be more relevant for these outcomes.

Despite some limitations, our study's strengths include its longitudinal design, large sample size, and robust statistical methods, offering a comprehensive view of the relationship between greenspace and child development. By adjusting for confounders, we highlight greenspace's role in shaping cognitive and behavioural outcomes during early childhood. Our findings stress the importance of a longitudinal approach, particularly regarding conduct problems and cognitive abilities. However, the mechanisms behind these effects are unclear and require further investigation. The relationship is likely influenced by factors like physical activity and social engagement, which may vary across developmental stages. Clinically, integrating green spaces into interventions, especially in early childhood, may support behavioural and cognitive development.

## Funding information

This work was supported by the Bloomsbury Colleges PhD studentship awarded to Georgia Cronshaw.

## Ethics statement

The Millennium Cohort Study (MCS)'s responsible authorities obtained ethical approval.

Parents and their children also gave written informed consent for their participation. For more information, please visit the MCS website: https://cls.ucl.ac.uk/cls.studies/millennium‐cohort‐study/.

## Conflict of interest statement

The authors declare that they have no conflicts of interest.

## Supporting information


**Table S1.** Model specification.
**Table S2.** Correlation of greenspace and subscales of the Strengths and Difficulties Questionnaire.
**Table S3.** Correlation of greenspace and cognitive ability.
**Table S4.** Model 0 ‐ Fixed and random effects estimates for emotional symptoms, peer problems, conduct problems, hyperactivity/inattention and cognitive ability (*n* = 6946).
**Table S5.** Model 1 ‐ Fixed and random effects estimates for emotional symptoms, peer problems, conduct problems, hyperactivity/inattention and cognitive ability (*n* = 6946).
**Table S6.** Model 2 ‐ Fixed and random effects estimates for emotional symptoms, peer problems, conduct problems, hyperactivity/inattention and cognitive ability (*n* = 6946).
**Table S7.** Model 3 ‐ Fixed and random effects estimates for emotional symptoms, peer problems, conduct problems, hyperactivity/inattention cognitive ability (*n* = 6946).
**Table S8.** Complete Case Analysis ‐ Fixed and random effects estimates for conduct problems and cognitive ability complete case.
**Table S9.** Fixed and random effects estimates for conduct problems and cognitive ability (including sex, greenspace interaction terms) (*n* = 6946).
**Table S10.** Fixed and random effects estimates for conduct problems and cognitive ability (including school type) (*n* = 6946).
**Table S11.** Fixed and random effects estimates for conduct problems and cognitive ability (including London resident status) (*n* = 6946).
**Table S12.** Fixed and random effects estimates for conduct problems and cognitive ability (replacing urban/rural area with settlement type) (*n* = 6946).
**Figure S1.** Analytic sample creation.
**Figure S2.** Predicted trajectories of cognitive ability score (A) and conduct problems (B) by greenspace decile from ages 3 to 11 years old with the addition school type (independent or state), using the growth curve models fixed effects.

## Data Availability

The data can be accessed from the UK Data Service at: http://doi.org/10.5255/UKDA‐Series‐2000031. Example code is available at: https://github.com/gcrons122/mcs_01_phd.
