# Supplementary material for: The role of neighbourhood greenspace quantity on mental health and cognitive development in early to middle childhood: a multilevel growth curve analysis of the UK Millennium Cohort Study
Source: Child Adolesc Ment Health. 2025 Mar 24;30(2):159–67. doi: 10.1111/camh.12767 (PMC12079720; doi:10.1111/camh.12767)
Supplement: Supplementary file 1 — Table S1. Model specification. Table S2. Correlation of greenspace and subscales of the Strengths and Difficulties Questionnaire. Table S3. Correlation of greenspace and cognitive ability. Table S4. Model 0 ‐ Fixed and random effects estimates for emotional symptoms, peer problems, conduct problems, hyperactivity/inattention and cognitive ability (n = 6946). Table S5. Model 1 ‐ Fixed and random effects estimates for emotional symptoms, peer problems, conduct problems, hyperactivity/inattention and cognitive ability (n = 6946). Table S6. Model 2 ‐ Fixed and random effects estimates for emotional symptoms, peer problems, conduct problems, hyperactivity/inattention and cognitive ability (n = 6946). Table S7. Model 3 ‐ Fixed and random effects estimates for emotional symptoms, peer problems, conduct problems, hyperactivity/inattention cognitive ability (n = 6946). Table S8. Complete Case Analysis ‐ Fixed and random effects estimates for conduct problems and cognitive ability complete case. Table S9. Fixed and random effects estimates for conduct problems and cognitive ability (including sex, greenspace interaction terms) (n = 6946). Table S10. Fixed and random effects estimates for conduct problems and cognitive ability (including school type) (n = 6946). Table S11. Fixed and random effects estimates for conduct problems and cognitive ability (including London resident status) (n = 6946). Table S12. Fixed and random effects estimates for conduct problems and cognitive ability (replacing urban/rural area with settlement type) (n = 6946). Figure S1. Analytic sample creation. Figure S2. Predicted trajectories of cognitive ability score (A) and conduct problems (B) by greenspace decile from ages 3 to 11 years old with the addition school type (independent or state), using the growth curve models fixed effects. [file CAMH-30-159-s001.docx]

**Supporting Information**

**The Role of Neighbourhood Greenspace Quantity on Mental Health and Cognitive Development in Early to Middle Childhood: A Multilevel Growth Curve Analysis of the UK Millennium Cohort Study**

Georgia Cronshaw BSc^1^, Emily Midouhas PhD^1^, Peninah Murage PhD^2^, Eirini Flouri PhD^1^

^1^Department of Psychology and Human Development, IOE, UCL’s Faculty of Education and Society, London, UK

^2^Public Health Environment and Society Department, London School of Hygiene and Tropical Medicine, London, UK

**Correspondence**

Georgia Cronshaw georgia.cronshaw.19@ucl.ac.uk

**Figure S1:** Analytic Sample Creation

MCS Initial Sample

n = 19,243

England only

n = 7,000

Strata: England Advantaged, Disadvantaged, Ethnic

n = 6,946

Analytic Sample

n = 6,946

| ***Table S1***  *Model Specification* |  |
| --- | --- |
| **Models** | **Variables** |
| Model 0 | Age + Age^2x^ |
| Model 1 | Model 0 + Study Design + Greenspace + Greenspace:Age |
| Model 2 | Model 1 + Neighbourhood Level Variables^a^ + Garden Access |
| Model 3 | Model 2 + Child Level Variables^b^ |
| Model 4 | Model 3 + Family Level Variables^c^ |

x Age^2^ in the SDQ models only

a Urbanicity/Rurality, Air Pollution

b Gender and Ethnicity

c Residential Mobility, Poverty Indicator, Maternal Education, Maternal Psychological Distress, Family Structure, Homeownership

| ***Table S2***  *Correlation of Greenspace and subscales of the Strengths and Difficulties Questionnaire* | | | | | | | | | | | | | | | | | | | | |
| --- | --- | --- | --- | --- | --- | --- | --- | --- | --- | --- | --- | --- | --- | --- | --- | --- | --- | --- | --- | --- |
|  | Variables | | | | | | | | | | | | | | | | | | | |
| Characteristics | 1. | 2. | 3. | 4. | 5. | 6. | 7. | 8. | 9. | 10. | 11. | 12. | 13. | 14. | 15. | 16. | 17. | 18. | 19. | 20. |
|  |  |  |  |  |  |  |  |  |  |  |  |  |  |  |  |  |  |  |  |  |
| 1. Greenspace (S2) | 1.000 |  |  |  |  |  |  |  |  |  |  |  |  |  |  |  |  |  |  |  |
| 2. Greenspace (S3) | 0.915 | 1.000 |  |  |  |  |  |  |  |  |  |  |  |  |  |  |  |  |  |  |
| 3. Greenspace (S4) | 0.864 | 0.933 | 1.000 |  |  |  |  |  |  |  |  |  |  |  |  |  |  |  |  |  |
| 4. Greenspace (S5) | 0.801 | 0.863 | 0.907 | 1.000 |  |  |  |  |  |  |  |  |  |  |  |  |  |  |  |  |
| 5. Emotional Symptoms (S2) | -0.087 | -0.088 | -0.089 | -0.087 | 1.000 |  |  |  |  |  |  |  |  |  |  |  |  |  |  |  |
| 6. Emotional Symptoms (S3) | -0.069 | -0.071 | -0.076 | -0.069 | 0.428 | 1.000 |  |  |  |  |  |  |  |  |  |  |  |  |  |  |
| 7. Emotional Symptoms (S4) | -0.081 | -0.082 | -0.085 | -0.070 | 0.361 | 0.503 | 1.000 |  |  |  |  |  |  |  |  |  |  |  |  |  |
| 8. Emotional Symptoms(S5) | ***-0.032*** | ***-0.036*** | -0.042 | ***-0.032*** | 0.261 | 0.377 | 0.482 | 1.000 |  |  |  |  |  |  |  |  |  |  |  |  |
| 9. Conduct Problems (S2) | -0.058 | -0.067 | -0.073 | -0.069 | 0.295 | 0.231 | 0.243 | 0.224 | 1.000 |  |  |  |  |  |  |  |  |  |  |  |
| 10. Conduct Problems (S3) | -0.064 | -0.074 | -0.081 | -0.077 | 0.191 | 0.303 | 0.273 | 0.247 | 0.504 | 1.000 |  |  |  |  |  |  |  |  |  |  |
| 11. Conduct Problems (S4) | -0.054 | -0.060 | -0.062 | -0.064 | 0.173 | 0.218 | 0.374 | 0.279 | 0.447 | 0.586 | 1.000 |  |  |  |  |  |  |  |  |  |
| 12. Conduct Problems (S5) | ***-0.036*** | ***-0.037*** | -0.045 | -0.044 | 0.152 | 0.184 | 0.239 | 0.382 | 0.392 | 0.486 | 0.564 | 1.000 |  |  |  |  |  |  |  |  |
| 13. Hyperactivity/inattention (S2) | -0.058 | -0.060 | -0.061 | -0.064 | 0.239 | 0.177 | 0.189 | 0.166 | 0.479 | 0.357 | 0.335 | 0.277 | 1.000 |  |  |  |  |  |  |  |
| 14. Hyperactivity/inattention (S3) | -0.068 | -0.067 | -0.066 | -0.065 | 0.169 | 0.262 | 0.229 | 0.222 | 0.363 | 0.522 | 0.424 | 0.362 | 0.570 | 1.000 |  |  |  |  |  |  |
| 15. Hyperactivity/inattention (S4) | -0.058 | -0.058 | -0.055 | -0.062 | 0.149 | 0.182 | 0.284 | 0.244 | 0.339 | 0.427 | 0.544 | 0.395 | 0.505 | 0.669 | 1.000 |  |  |  |  |  |
| 16. Hyperactivity/inattention (S5) | ***-0.039*** | -0.044 | ***-0.036*** | ***-0.040*** | 0.138 | 0.159 | 0.225 | 0.369 | 0.324 | 0.393 | 0.456 | 0.544 | 0.433 | 0.559 | 0.661 | 1.000 |  |  |  |  |
| 17. Peer Problems (S2) | -0.081 | -0.090 | -0.098 | -0.100 | 0.335 | 0.271 | 0.249 | 0.211 | 0.258 | 0.195 | 0.187 | 0.159 | 0.245 | 0.218 | 0.206 | 0.175 | 1.000 |  |  |  |
| 18. Peer Problems (S3) | -0.113 | -0.117 | -0.127 | -0.118 | 0.249 | 0.388 | 0.306 | 0.247 | 0.204 | 0.284 | 0.225 | 0.197 | 0.205 | 0.288 | 0.251 | 0.234 | 0.405 | 1.000 |  |  |
| 19. Peer Problems(S4) | -0.112 | -0.109 | -0.113 | -0.102 | 0.207 | 0.269 | 0.420 | 0.291 | 0.212 | 0.278 | 0.347 | 0.252 | 0.215 | 0.270 | 0.321 | 0.286 | 0.353 | 0.528 | 1.000 |  |
| 20. Peer Problems (S5) | -0.062 | -0.055 | -0.056 | -0.055 | 0.168 | 0.223 | 0.285 | 0.465 | 0.203 | 0.238 | 0.282 | 0.361 | 0.198 | 0.268 | 0.291 | 0.365 | 0.293 | 0.386 | 0.494 | 1.000 |
| All Pearson’s correlations are statistically significant at p < 0.001, with the exception of those in ***bold and italicised***, which are significant at p < 0.01. | | | | | | | | | | | | | | | | | | | | |

| ***Table S3***  *Correlation of Greenspace and Cognitive Ability* | | | | | | | | |
| --- | --- | --- | --- | --- | --- | --- | --- | --- |
|  | Variables | | | | | | | |
| Characteristics | 1. | 2. | 3. | 4. | 5. | 6. | 7. | 8. |
|  |  |  |  |  |  |  |  |  |
| 1. Greenspace (S2) | 1.000 |  |  |  |  |  |  |  |
| 2. Greenspace (S3) | 0.915 | 1.000 |  |  |  |  |  |  |
| 3. Greenspace (S4) | 0.864 | 0.933 | 1.000 |  |  |  |  |  |
| 4. Greenspace (S5) | 0.801 | 0.863 | 0.907 | 1.000 |  |  |  |  |
| 5. Cognitive Ability (S2) | 0.212 | 0.216 | 0.222 | 0.222 | 1.000 |  |  |  |
| 6. Cognitive Ability (S3) | 0.148 | 0.150 | 0.154 | 0.152 | 0.542 | 1.000 |  |  |
| 7. Cognitive Ability (S4) | 0.086 | 0.087 | 0.083 | 0.087 | 0.486 | 0.611 | 1.000 |  |
| 8. Cognitive Ability (S5) | 0.075 | 0.074 | 0.075 | 0.077 | 0.381 | 0.463 | 0.523 | 1.000 |
| All correlations are statistically significant at p < 0.001. | | | | | | | | |

| ***Table S4***  *Fixed and Random Effects Estimates for Emotional Symptoms, Peer Problems, Conduct Problems, Hyperactivity and Inattention Symptoms and Cognitive Ability (n = 6946)* | | | | | | | | | | | | | | |
| --- | --- | --- | --- | --- | --- | --- | --- | --- | --- | --- | --- | --- | --- | --- |
| **Model 0** | Emotional Symptoms | |  | Peer Problems | |  | Conduct Problems | |  | Hyperactivity/Inattention Symptoms | |  | Cognitive Ability | |
|  | *b (SE)* | *p* |  | *b (SE)* | *p* |  | *b (SE)* | *p* |  | *b (SE)* | *p* |  | *b (SE)* | *p* |
| **Fixed Effects** |  |  |  |  |  |  |  |  |  |  |  |  |  |  |
| Age | **0.065***** (0.006) | 0.000 |  | **-0.030***** (0.004) | 0.000 |  | **-0.215***** (0.005) | 0.000 |  | **-0.107***** (0.006) | 0.000 |  | 0.034 (0.052) | 0.515 |
| Age^2^ | **0.006***** (0.001) | 0.000 |  | **0.018***** (0.001) | 0.000 |  | **0.053***** (0.001) | 0.000 |  | **0.017***** (0.002) | 0.000 |  | - | - |
| Constant | **1.524***** (0.029) | 0.000 |  | **1.198***** (0.030) | 0.000 |  | **1.348***** (0.029) | 0.000 |  | **3.317***** (0.044) | 0.000 |  | **100.710***** (0.0351) | 0.000 |
| **Random Effects** |  |  |  |  |  |  |  |  |  |  |  |  |  |  |
| **Level 2 (child)** |  |  |  |  |  |  |  |  |  |  |  |  |  |  |
| Between-child intercept variance | 1.258 (0.046) |  |  | 1.036 (0.044) |  |  | 1.384 (0.049) |  |  | 3.339 (0.088) |  |  | 99.340 (3.560) |  |
| Between-child slope variance | 0.029 (0.002) |  |  | 0.019 (0.002) |  |  | 0.019 (0.001) |  |  | 0.035 (0.002) |  |  | 1.351 (0.108) |  |
| Between-child intercept/slope variance covariance | 0.093 (0.008) |  |  | 0.035 (0.005) |  |  | -0.051 (0.006) |  |  | 0.042 (0.010) |  |  | -0.261 (0.451) |  |
| **Level 3 (area)** |  |  |  |  |  |  |  |  |  |  |  |  |  |  |
| Variance | 0.068 (0.012) |  |  | 0.095 (0.012) |  |  | 0.106 (0.019) |  |  | 0.155 (0.028) |  |  | 19.312 (2.343) |  |
| **Residual variance (level 1)** | 1.477 (0.046) |  |  | 1.261 (0.036) |  |  | 1.284 (0.031) |  |  | 2.153 (0.044) |  |  | 89.836 (2.049) |  |
| **ICC Level 2**  **ICC Level 3** | 0.449  0.024 |  |  | 0.433  0.040 |  |  | 0.500  0.038 |  |  | 0.590  0.027 |  |  | 0.476  0.093 |  |
| **Note.** $\boldsymbol{b}$ **= unstandardised coefficients; SE = Standard Error. *p < .05. **p < .01. ***p < .001** | | | | | | | | | | | | | | |

| ***Table S5***  *Fixed and Random Effects Estimates for Emotional Symptoms, Peer Problems, Conduct Problems, Hyperactivity and Inattention Symptoms and Cognitive Ability (n = 6946)* | | | | | | | | | | | | | | |
| --- | --- | --- | --- | --- | --- | --- | --- | --- | --- | --- | --- | --- | --- | --- |
| **Model 1** | Emotional Symptoms | |  | Peer Problems | |  | Conduct Problems | |  | Hyperactivity and Inattention Symptoms | |  | Cognitive Ability | |
|  | *b (SE)* | *p* |  | *b (SE)* | *p* |  | *b (SE)* | *p* |  | *b (SE)* | *p* |  | *b (SE)* | *p* |
| **Fixed Effects** |  |  |  |  |  |  |  |  |  |  |  |  |  |  |
| Age | **0.066***** (0.009) | 0.000 |  | **-0.030***** (0.007) | 0.000 |  | **-0.227***** (0.007) | 0.000 |  | **-0.115***** (0.010) | 0.000 |  | **0.459***** (0.108) | 0.000 |
| Age^2^ | **0.055***** (0.001) | 0.000 |  | **0.018***** (0.001) | 0.000 |  | **0.053***** (0.001) | 0.000 |  | **0.017***** (0.002) | 0.000 |  | - | - |
| Greenspace | **-0.018*** (0.007) | 0.014 |  | -0.011 (0.007) | 0.137 |  | -0.015 (0.008) | 0.058 |  | -0.009 (0.010) | 0.361 |  | 0.132 (0.074) | 0.075 |
| Greenspace x Age | -0.000 (0.002) | 0.961 |  | -0.000 (0.001) | 0.969 |  | **0.003*** (0.001) | 0.025 |  | 0.002 (0.002) | 0.296 |  | **-0.096***** (0.020) | 0.000 |
| England Disadvantaged (ref. England advantaged) | **0.320***** (0.044) | 0.000 |  | **0.396***** (0.044) | 0.000 |  | **0.464***** (0.055) | 0.000 |  | **0.493***** (0.070) | 0.000 |  | **-4.691***** (0.596) | 0.000 |
| England Ethnic (ref. England advantaged) | **0.494***** (0.089) | 0.000 |  | **0.682***** (0.063) | 0.000 |  | **0.263**** (0.078) | 0.001 |  | **0.470***** (0.125) | 0.000 |  | **-8.661***** (0.986) | 0.000 |
| Constant | **1.460***** (0.050) | 0.000 |  | **1.058***** (0.050) | 0.000 |  | **1.234***** (0.058) | 0.000 |  | **3.156***** (0.072) | 0.000 |  | **102.456***** (0.586) | 0.000 |
| **Random Effects** |  |  |  |  |  |  |  |  |  |  |  |  |  |  |
| **Level 2 (child)** |  |  |  |  |  |  |  |  |  |  |  |  |  |  |
| Between-child intercept variance | 1.256 (0.046) |  |  | 1.034 (0.043) |  |  | 1.381 (0.048) |  |  | 3.334 (0.088) |  |  | 99.216 (3.557) |  |
| Between-child slope variance | 0.029 (0.002) |  |  | 0.019 (0.002) |  |  | 0.019 (0.001) |  |  | 0.035 (0.002) |  |  | 1.291 (0.103) |  |
| Between-child intercept/slope variance covariance | 0.094 (0.008) |  |  | 0.038 (0.005) |  |  | -0.051 (0.006) |  |  | 0.043 (0.010) |  |  | -0.018 (0.410) |  |
| **Level 3 (area)** |  |  |  |  |  |  |  |  |  |  |  |  |  |  |
| Variance | 0.032 (0.008) |  |  | 0.038 (0.009) |  |  | 0.055 (0.018) |  |  | 0.098 (0.024) |  |  | 10.031 (1.436) |  |
| **Residual variance (level 1)** | 1.477 (0.046) |  |  | 1.262 (0.036) |  |  | 1.285 (0.031) |  |  | 2.153 (0.044) |  |  | 89.886 (2.044) |  |
| **Note.** $\boldsymbol{b}$ **= unstandardised coefficients; SE = Standard Error. *p < .05. **p < .01. ***p < .001** | | | | | | | | | | | | | | |

| ***Table S6***  *Fixed and Random Effects Estimates for Emotional Symptoms, Peer Problems, Conduct Problems, Hyperactivity and Inattention Symptoms and Cognitive Ability (n = 6946)* | | | | | | | | | | | | | | |
| --- | --- | --- | --- | --- | --- | --- | --- | --- | --- | --- | --- | --- | --- | --- |
| **Model 2** | Emotional Symptoms | |  | Peer Problems | |  | Conduct Problems | |  | Hyperactivity and Inattention Symptoms | |  | Cognitive Ability | |
|  | *b (SE)* | *p* |  | *b (SE)* | *p* |  | *b (SE)* | *p* |  | *b (SE)* | *p* |  | *b (SE)* | *p* |
| **Fixed Effects** |  |  |  |  |  |  |  |  |  |  |  |  |  |  |
| Age | **0.067***** (0.010) | 0.000 |  | **-0.029***** (0.007) | 0.000 |  | **-0.227***** (0.007) | 0.000 |  | **-0.114***** (0.010) | 0.000 |  | **0.451***** (0.108) | 0.000 |
| Age^2^ | **0.006***** (0.001) | 0.000 |  | **0.018***** (0.001) | 0.000 |  | **0.053***** (0.001) | 0.000 |  | **0.017***** (0.002) | 0.000 |  | - | - |
| Greenspace | -0.008 (0.010) | 0.393 |  | -0.002 (0.010) | 0.866 |  | -0.008 (0.011) | 0.451 |  | 0.002 (0.014) | 0.866 |  | 0.058 (0.102) | 0.571 |
| Greenspace x Age | -0.000 (0.002) | 0.936 |  | -0.000 (0.001) | 0.894 |  | **0.003* (**0.001) | 0.031 |  | 0.002 (0.002) | 0.337 |  | **-0.096***** (0.020) | 0.000 |
| England Disadvantaged (ref. England advantaged) | **0.302***** (0.046) | 0.000 |  | **0.375***** (0.043) | 0.000 |  | **0.445***** (0.055) | 0.000 |  | **0.462***** (0.072) | 0.000 |  | **-4.522***** (0.596) | 0.000 |
| England Ethnic (ref. England advantaged) | **0.465***** (0.098) | 0.000 |  | **0.615***** (0.073) | 0.000 |  | **0.234**** (0.088) | 0.008 |  | **0.402**** (0.143) | 0.005 |  | **-8.188***** (1.020) | 0.000 |
| Living in an Urban Area | 0.091 (0.063) | 0.151 |  | 0.009 (0.060) | 0.886 |  | 0.096 (0.064) | 0.130 |  | 0.092 (0.095) | 0.334 |  | -0.340 (0.615) | 0.581 |
| Air Pollution (PM10) | 0.003 (0.011) | 0.803 |  | 0.015 (0.010) | 0.134 |  | -0.006 (0.012) | 0.612 |  | 0.004 (0.015) | 0.794 |  | 0.084 (0.090) | 0.347 |
| Sole Access to a Garden | -0.178 (0.106) | 0.093 |  | **-0.255***** (0.054) | 0.000 |  | **-0.240*** (0.104) | 0.021 |  | **-0.371**** (0.129) | 0.004 |  | **1.892**** (0.564) | 0.001 |
| Constant | **1.488***** (0.142) | 0.000 |  | **1.147***** (0.144) | 0.000 |  | **1.387***** (0.176) | 0.000 |  | **3.325***** (0.196) | 0.000 |  | **101.882***** (1.319) | 0.000 |
| **Random Effects** |  |  |  |  |  |  |  |  |  |  |  |  |  |  |
| **Level 2 (child)** |  |  |  |  |  |  |  |  |  |  |  |  |  |  |
| Between-child intercept variance | 1.255 (0.046) |  |  | 1.027 (0.043) |  |  | 1.375 (0.047) |  |  | 3.323 (0.087) |  |  | 98.618 (3.524) |  |
| Between-child slope variance | 0.029 (0.002) |  |  | 0.019 (0.002) |  |  | 0.019 (0.001) |  |  | 0.035 (0.002) |  |  | 1.293 (0.102) |  |
| Between-child intercept/slope variance covariance | 0.094 (0.008) |  |  | 0.036 (0.005) |  |  | -0.050 (0.006) |  |  | 0.044 (0.010) |  |  | -0.008 (0.407) |  |
| **Level 3 (area)** |  |  |  |  |  |  |  |  |  |  |  |  |  |  |
| Variance | 0.032 (0.008) |  |  | 0.038 (0.009) |  |  | 0.053 (0.016) |  |  | 0.102 (0.024) |  |  | 9.937 (1.427) |  |
| **Residual variance (level 1)** | 1.476 (0.046) |  |  | 1.262 (0.036) |  |  | 1.283 (0.031) |  |  | 2.150 (0.044) |  |  | 89.925 (2.040) |  |
| **Note.** $\boldsymbol{b}$ **= unstandardised coefficients; SE = Standard Error. *p < .05. **p < .01. ***p < .001** | | | | | | | | | | | | | | |

| ***Table S7***  *Fixed and Random Effects Estimates for Emotional Symptoms, Peer Problems, Conduct Problems, Hyperactivity and Inattention Symptoms and Cognitive Ability (n = 6946)* | | | | | | | | | | | | | | |
| --- | --- | --- | --- | --- | --- | --- | --- | --- | --- | --- | --- | --- | --- | --- |
| **Model 3** | Emotional Symptoms | |  | Peer Problems | |  | Conduct Problems | |  | Hyperactivity/Inattention Symptoms | |  | Cognitive Ability | |
|  | *b (SE)* | *p* |  | *b (SE)* | *p* |  | *b (SE)* | *p* |  | *b (SE)* | *p* |  | *b (SE)* | *p* |
| **Fixed Effects** |  |  |  |  |  |  |  |  |  |  |  |  |  |  |
| Age | **0.066***** (0.010) | 0.000 |  | **-0.029***** (0.007) | 0.000 |  | **-0.226***** (0.007) | 0.000 |  | **-0.114***** (0.010) | 0.000 |  | **0.460***** (0.108) | 0.000 |
| Age^2^ | **0.006***** (0.001) | 0.000 |  | **0.018***** (0.001) | 0.000 |  | **0.053***** (0.001) | 0.000 |  | **0.017***** (0.002) | 0.000 |  | - | - |
| Greenspace | -0.007 (0.010) | 0.467 |  | -0.001 (0.010) | 0.899 |  | -0.009 (0.010) | 0.399 |  | 0.002 (0.014) | 0.902 |  | 0.015 (0.101) | 0.882 |
| Greenspace x Age | -0.000 (0.002) | 0.972 |  | -0.000 (0.001) | 0.951 |  | **0.003*** (0.001) | 0.034 |  | 0.002 (0.002) | 0.333 |  | **-0.097***** (0.020) | 0.000 |
| England Disadvantaged (ref. England advantaged) | **0.283***** (0.044) | 0.000 |  | **0.345***** (0.043) | 0.000 |  | **0.447***** (0.053) | 0.000 |  | **0.455***** (0.068) | 0.000 |  | **-4.068***** (0.589) | 0.000 |
| England Ethnic (ref. England advantaged) | **0.276**** (0.103) | 0.007 |  | **0.293***** (0.079) | 0.000 |  | **0.338**** (0.101) | 0.001 |  | **0.406**** (0.153) | 0.008 |  | **-4.251***** (1.001) | 0.000 |
| Living in an Urban Area | 0.093 (0.063) | 0.144 |  | 0.025 (0.061) | 0.676 |  | 0.098 (0.063) | 0.119 |  | -0.101 (0.092) | 0.269 |  | -0.517 (0.608) | 0.395 |
| Air Pollution (PM10) | -0.001 (0.010) | 0.955 |  | 0.006 (0.010) | 0.511 |  | -0.003 (0.012) | 0.793 |  | 0.005 (0.015) | 0.744 |  | 0.001 (0.095) | 0.989 |
| Sole Access to a Garden | -0.182 (0.108) | 0.093 |  | **-0.240***** (0.054) | 0.000 |  | **-0.251*** (0.103) | 0.014 |  | **-0.381**** (0.128) | 0.003 |  | **1.606**** (0.549) | 0.003 |
| Female | -0.038 (0.032) | 0.229 |  | **-0.233***** (0.027) | 0.000 |  | **-0.325***** (0.035) | 0.000 |  | **-0.744***** (0.051) | 0.000 |  | **1.910***** (0.308) | 0.000 |
| Ethnicity Mixed (ref. Ethnicity White) | 0.130 (0.105) | 0.215 |  | 0.142 (0.087) | 0.103 |  | 0.005 (0.093) | 0.958 |  | -0.033 (0.149) | 0.827 |  | -1.030 (1.046) | 0.325 |
| Ethnicity Indian (ref. Ethnicity White) | 0.039 (0.126) | 0.758 |  | **0.391***** (0.106) | 0.000 |  | -0.211 (0.114) | 0.064 |  | -0.177 (0.222) | 0.426 |  | -1.513 (1.284) | 0.238 |
| Ethnicity Pakistani/Bangladeshi (ref. Ethnicity White) | **0.627***** (0.126) | 0.000 |  | **0.727***** (0.080) | 0.000 |  | -0.056 (0.078) | 0.468 |  | **-0.289* (0.123)** | 0.019 |  | **-9.261***** (0.806) | 0.000 |
| Ethnicity Black (ref. Ethnicity White) | -0.108 (0.137) | 0.431 |  | 0.211 (0.150) | 0.160 |  | -0.238 (0.159) | 0.134 |  | -0.377 (0.202) | 0.062 |  | **-6.245***** (1.535) | 0.000 |
| Ethnicity Other (ref. Ethnicity White) | 0.140 (0.138) | 0.308 |  | **0.390*** (0.178) | 0.028 |  | **-0.369**** (0.116) | 0.001 |  | -0.159 (0.209) | 0.445 |  | **-3.072*** (1.046) | 0.003 |
| Constant | **1.477***** (0.141) | 0.000 |  | 1.251*** (0.144) | 0.000 |  | **1.551***** (0.177) | 0.000 |  | **3.715***** (0.196) | 0.000 |  | **101.233***** (1.343) | 0.000 |
| **Random Effects** |  |  |  |  |  |  |  |  |  |  |  |  |  |  |
| **Level 2 (child)** |  |  |  |  |  |  |  |  |  |  |  |  |  |  |
| Between-child intercept variance | 1.248 (0.046) |  |  | 1.002 (0.043) |  |  | 1.349 (0.46) |  |  | 3.180 (0.084) |  |  | 95.227 (3.325) |  |
| Between-child slope variance | 0.030 (0.002) |  |  | 0.019 (0.002) |  |  | 0.019 (0.001) |  |  | 0.035 (0.002) |  |  | 1.287 (0.102) |  |
| Between-child intercept/slope variance covariance | 0.095 (0.008) |  |  | 0.038 (0.005) |  |  | -0.051 (0.006) |  |  | 0.037 (0.010) |  |  | 0.689 (0.399) |  |
| **Level 3 (area)** |  |  |  |  |  |  |  |  |  |  |  |  |  |  |
| Variance | 0.027 (0.008) |  |  | 0.036 (0.009) |  |  | 0.046 (0.015) |  |  | 0.088 (0.021) |  |  | 9.165 (1.356) |  |
| **Residual variance (level 1)** | 1.475 (0.045) |  |  | 1.261 (0.036) |  |  | 1.283 (0.031) |  |  | 2.150 (0.044) |  |  | 89.947 (2.047) |  |
| **Note.** $\boldsymbol{b}$ **= unstandardised coefficients; SE = Standard Error. *p < .05. **p < .01. ***p < .001** | | | | | | | | | | | | | | |

| ***Table S8***  *Fixed and Random Effects Estimates for Conduct Problems and Cognitive Ability Complete Case* | | | | | |
| --- | --- | --- | --- | --- | --- |
|  | Conduct Problems (n=5944) | |  | Cognitive Ability (n = 5942) | |
|  | *b (SE)* | *p* |  | *b (SE)* | *p* |
| **Fixed Effects** |  |  |  |  |  |
| Age | **-0.228***** (0.008) | 0.000 |  | **0.254**** (0.095) | 0.008 |
| Age^2^ | **0.054***** (0.001) | 0.000 |  | **-** | - |
| Greenspace | -0.004 (0.010) | 0.712 |  | -0.129 (0.093) | 0.167 |
| Greenspace x Age | **0.003*** (0.001) | 0.013 |  | **-0.070***** (0.018) | 0.000 |
| England Disadvantaged (ref. England advantaged) | **0.187***** (0.047) | 0.000 |  | **-1.704**** (0.502) | 0.001 |
| England Ethnic (ref. England advantaged) | 0.031 (0.080) | 0.703 |  | **-2.017*** (0.848) | 0.017 |
| Living in an Urban Area | 0.093 (0.063) | 0.140 |  | -0.919 (0.574) | 0.109 |
| Air Pollution (PM10) | 0.002 (0.013) | 0.904 |  | -0.007 (0.085) | 0.932 |
| Sole Access to a Garden | -0.064 (0.112) | 0.564 |  | -0.410 (0.636) | 0.520 |
| Female | **-0.327***** (0.034) | 0.000 |  | **1.937***** (0.304) | 0.000 |
| Ethnicity Mixed (ref. Ethnicity White) | -0.135 (0.085) | 0.111 |  | 0.290 (1.065) | 0.785 |
| Ethnicity Indian (ref. Ethnicity White) | -0.049 (0.113) | 0.660 |  | -1.047 (1.132) | 0.355 |
| Ethnicity Pakistani/Bangladeshi (ref. Ethnicity White) | -0.086 (0.093) | 0.354 |  | **-6.548***** (1.096) | 0.000 |
| Ethnicity Black (ref. Ethnicity White) | **-0.471***** (0.120) | 0.000 |  | **-3.067**** (1.137) | 0.007 |
| Ethnicity Other (ref. Ethnicity White) | **-0.404**** (0.149) | 0.007 |  | -0.777 (1.610) | 0.629 |
| Moved House | -0.009 (0.031) | 0.773 |  | -0.066 (0.307) | 0.829 |
| Below the poverty line | **0.186***** (0.059) | 0.002 |  | **-3.148***** (0.462) | 0.000 |
| Lives with both natural parents | **-0.242 **** (0.072) | 0.001 |  | 0.886 (0.529) | 0.094 |
| Owns Home | **-0.418***** (0.053) | 0.000 |  | **3.393***** (0.433) | 0.000 |
| Mother University Educated | **-0.293***** (0.048) | 0.000 |  | **5.756***** (0.404) | 0.000 |
| Maternal Psychological Distress | **0.079***** (0.006) | 0.000 |  | **-0.126**** (0.047) | 0.007 |
| Constant | **1.588***** (0.218) | 0.000 |  | **100.648***** (1.314) | 0.000 |
| **Random Effects** |  |  |  |  |  |
| **Level 2 (child)** |  |  |  |  |  |
| Between-child intercept variance | 1.107 (0.041) |  |  | 79.056 (2.894) |  |
| Between-child slope variance | 0.019 (0.001) |  |  | 1.121 (0.100) |  |
| Between-child intercept/slope variance covariance | -0.038 (0.006) |  |  | 1.222 (0.322) |  |
| **Level 3 (area)** |  |  |  |  |  |
| Variance | 0.025 (0.013) |  |  | 4.848 (0.818) |  |
| **Residual variance (level 1)** | 1.258 (0.031) |  |  | 88.513 (2.108) |  |
| **Note.** $\boldsymbol{b}$ **= unstandardised coefficients; SE = Standard Error. *p < .05. **p < .01. ***p < .001** | | | | | |

| ***Table S9***  *Fixed and Random Effects Estimates for Conduct Problems and Cognitive Ability (including sex, greenspace interaction terms (n = 6946)* | | | | | |
| --- | --- | --- | --- | --- | --- |
|  | Conduct Problems | |  | Cognitive Ability | |
|  | *b (SE)* | *p* |  | *b (SE)* | *p* |
| **Fixed Effects** |  |  |  |  |  |
| Age | **-0.233***** (0.011) | 0.000 |  | 0.085 (0.014) | 0.000 |
| Age^2^ | **0.053***** (0.001) | 0.000 |  | **-** | - |
| Greenspace | -0.003 (0.012) | 0.787 |  | -0.081 (0.105) | 0.437 |
| Greenspace x Age | **0.005**** (0.002) | 0.009 |  | **-0.133***** (0.022) | 0.000 |
| England Disadvantaged (ref. England advantaged) | **0.213***** (0.044) | 0.000 |  | **-1.701***** (0.480) | 0.000 |
| England Ethnic (ref. England advantaged) | 0.041 (0.090) | 0.088 |  | **-1.844*** (0.850) | 0.030 |
| Living in an Urban Area | 0.111 (0.058) | 0.053 |  | -0.511 (0.555) | 0.357 |
| Air Pollution (PM10) | -0.001 (0.058) | 0.928 |  | -0.021 (0.085) | 0.804 |
| Sole Access to a Garden | -0.091 (0.101) | 0.366 |  | 0.084 (0.541) | 0.877 |
| Female | **-0.381***** (0.062) | 0.000 |  | **1.864*** (0.504)** | 0.000 |
| Ethnicity Mixed (ref. Ethnicity White) | -0.139 (0.084) | 0.099 |  | 0.125 (0.975) | 0.898 |
| Ethnicity Indian (ref. Ethnicity White) | -0.105 (0.111) | 0.342 |  | **-2.551*** (1.212) | 0.035 |
| Ethnicity Pakistani/Bangladeshi (ref. Ethnicity White) | -0.156 (0.081) | 0.054 |  | **-8.195***** (0.853) | 0.000 |
| Ethnicity Black (ref. Ethnicity White) | **-0.491**** (0.148) | 0.001 |  | **-4.293**** (1.243) | 0.001 |
| Ethnicity Other (ref. Ethnicity White) | **-0.500***** (0.115) | 0.000 |  | **-2.196*** (1.091) | 0.044 |
| Moved House | -0.028 (0.029) | 0.347 |  | -0.149 (0.295) | 0.613 |
| Below the poverty line | **0.197***** (0.054) | 0.000 |  | **-3.429***** (0.422) | 0.000 |
| Lives with both natural parents | **-0.240***** (0.068) | 0.000 |  | 0.664 (0.490) | 0.176 |
| Owns Home | **-0.389***** (0.049) | 0.000 |  | **3.375***** (0.415) | 0.000 |
| Mother University Educated | **-0.302***** (0.048) | 0.000 |  | **5.998***** (0.383) | 0.000 |
| Maternal Psychological Distress | **0.080***** (0.005) | 0.000 |  | **-0.140**** (0.046) | 0.002 |
| Female x Age | 0.009 (0.013) | 0.510 |  | **-0.751***** (0.114) | 0.000 |
| Female x Greenspace | 0.013 (0.012) | 0.271 |  | -0.006 (0.097) | 0.955 |
| Female x Greenspace x Age | -0.004 (0.003) | 0.144 |  | **0.069**** (0.023) | 0.003 |
| Constant | **1.630***** (0.193) | 0.000 |  | **99.900***** (1.302) | 0.000 |
| **Random Effects** |  |  |  |  |  |
| **Level 2 (child)** |  |  |  |  |  |
| Between-child intercept variance | 1.128 (0.039) |  |  | 81.794 (2.906) |  |
| Between-child slope variance | 0.019 (0.001) |  |  | 1.224 (0.101) |  |
| Between-child intercept/slope variance covariance | -0.041 (0.006) |  |  | 0.929 (0.345) |  |
| **Level 3 (area)** |  |  |  |  |  |
| Variance | 0.020 (0.011) |  |  | 4.283 (0.718) |  |
| **Residual variance (level 1)** | 1.284 (0.031) |  |  | 90.075 (2.059) |  |
| **Note.** $\boldsymbol{b}$ **= unstandardised coefficients; SE = Standard Error. *p < .05. **p < .01. ***p < .001** | | | | | |

| ***Table S10***  *Fixed and Random Effects Estimates for Conduct Problems and Cognitive Ability (including school type) (n = 6946)* | | | | | |
| --- | --- | --- | --- | --- | --- |
|  | Conduct Problems | |  | Cognitive Ability | |
|  | *b (SE)* | *p* |  | *b (SE)* | *p* |
| **Fixed Effects** |  |  |  |  |  |
| Age | **-0.055***** (0.010) | 0.000 |  | 0.162 (0.129) | 0.208 |
| Age^2^ | **0.008***** (0.002) | 0.000 |  | **-** | - |
| Greenspace | -0.008 (0.011) | 0.480 |  | -0.082 (0.109) | 0.454 |
| Greenspace x Age | **0.003*** (0.001) | 0.019 |  | **-0.055*** (0.024) | 0.020 |
| England Disadvantaged (ref. England advantaged) | **0.183***** (0.045) | 0.000 |  | **-1.556**** (0.529) | 0.003 |
| England Ethnic (ref. England advantaged) | 0.019 (0.085) | 0.822 |  | -1.144 (0.914) | 0.211 |
| Living in an Urban Area | 0.091 (0.058) | 0.114 |  | -0.971 (0.62) | 0.118 |
| Air Pollution (PM10) | -0.008 (0.012) | 0.536 |  | -0.016 (0.099) | 0.875 |
| Sole Access to a Garden | -0.085 (0.093) | 0.362 |  | 0.160 (0.575) | 0.780 |
| Female | **-0.348***** (0.033) | 0.000 |  | **1.324***** (0.284) | 0.000 |
| Ethnicity Mixed (ref. Ethnicity White) | -0.113 (0.084) | 0.181 |  | 0.031 (1.074) | 0.977 |
| Ethnicity Indian (ref. Ethnicity White) | -0.097 (0.103) | 0.346 |  | -1.557 (1.239) | 0.209 |
| Ethnicity Pakistani/Bangladeshi (ref. Ethnicity White) | -0.128 (0.082) | 0.119 |  | **-6.527***** (0.907) | 0.000 |
| Ethnicity Black (ref. Ethnicity White) | **-0.440**** (0.138) | 0.001 |  | **-3.165*** (1.262) | 0.012 |
| Ethnicity Other (ref. Ethnicity White) | **-0.431**** (0.126) | 0.001 |  | -0.862 (1.013) | 0.395 |
| Moved House | **0.069*** (0.029) | 0.015 |  | -0.225 (0.303) | 0.457 |
| Below the poverty line | **0.189**** (0.058) | 0.001 |  | **-2.778***** (0.446) | 0.000 |
| Lives with both natural parents | **-0.231**** (0.070) | 0.001 |  | 0.505 (0.516) | 0.328 |
| Owns Home | **-0.353***** (0.050) | 0.000 |  | **3.163***** (0.431) | 0.000 |
| Mother University Educated | **-0.281***** (0.047) | 0.000 |  | **5.919***** (0.381) | 0.000 |
| Maternal Psychological Distress | **0.068***** (0.006) | 0.000 |  | **-0.131**** (0.050) | 0.009 |
| Fee-Paying School | -0.082 (0.049) | 0.093 |  | **2.488***** (0.631) | 0.000 |
| Constant | **1.834***** (0.210) | 0.000 |  | **100.393***** (1.440) | 0.000 |
| **Random Effects** |  |  |  |  |  |
| **Level 2 (child)** |  |  |  |  |  |
| Between-child intercept variance | 1.123 (0.046) |  |  | 91.466 (3.787) |  |
| Between-child slope variance | 0.018 (0.002) |  |  | 1.856 (0.228) |  |
| Between-child intercept/slope variance covariance | -0.005 (0.006) |  |  | -1.565 (0.605) |  |
| **Level 3 (area)** |  |  |  |  |  |
| Variance | 0.021 (0.013) |  |  | 5.744 (1.067) |  |
| **Residual variance (level 1)** | 0.895 (0.031) |  |  | 81.401 (2.370) |  |
| **Note.** $\boldsymbol{b}$**= unstandardised coefficients; SE = Standard Error. *p < .05. **p < .01. ***p < .001** | | | | | |
|  | | | | | |
| **Table S11**  *Fixed and Random Effects Estimates for Conduct Problems and Cognitive Ability (including London resident status) (n = 6946)* | | | | | |
|  | Conduct Problems | |  | Cognitive Ability | |
|  | *b (SE)* | *p* |  | *b (SE)* | *p* |
| **Fixed Effects** |  |  |  |  |  |
| Age | **-0.229*** (0.007)** | 0.000 |  | **0.479***** (0.109) | 0.000 |
| Age^2^ | **0.053*** (0.001)** | 0.000 |  | **-** | - |
| Greenspace | 0.000 (0.010) | 0.981 |  | -0.064 (0.089) | 0.473 |
| Greenspace x Age | **0.003* (0.001)** | 0.012 |  | **-0.099***** (0.020) | 0.000 |
| England Disadvantaged (ref. England advantaged) | **0.211***** (0.043) | 0.000 |  | **-1.685***** (0.470) | 0.000 |
| England Ethnic (ref. England advantaged) | 0.053 (0.084) | 0.532 |  | -2.019* (0.807) | 0.012 |
| Living in an Urban Area | 0.100 (0.057) | 0.081 |  | -0.453 (0.560) | 0.419 |
| Air Pollution (PM10) | 0.006 (0.012) | 0.628 |  | -0.079 (0.086) | 0.360 |
| Sole Access to a Garden | -0.110 (0.102) | 0.281 |  | 0.193 (0.543) | 0.722 |
| Female | **-0.331***** (0.032) | 0.000 |  | 1.928*** (0.272) | 0.000 |
| Ethnicity Mixed (ref. Ethnicity White) | -0.123 (0.084) | 0.143 |  | 0.026 (0.982) | 0.979 |
| Ethnicity Indian (ref. Ethnicity White) | -0.083 (0.11) | 0.454 |  | -2.662* (1.210) | 0.028 |
| Ethnicity Pakistani/Bangladeshi (ref. Ethnicity White) | **-0.157*** (0.080) | 0.049 |  | -8.204*** (0.835) | 0.000 |
| Ethnicity Black (ref. Ethnicity White) | **-0.431***** (0.149) | 0.004 |  | -4.655*** (1.287) | 0.000 |
| Ethnicity Other (ref. Ethnicity White) | **-0.455***** (0.118) | 0.000 |  | -2.473* (1.088) | 0.023 |
| Moved House | -0.030 (0.029) | 0.303 |  | -0.165 (0.294) | 0.576 |
| Below the poverty line | **0.193***** (0.055) | 0.000 |  | -3.413*** (0.421) | 0.000 |
| Lives with both natural parents | **-0.239***** (0.068) | 0.000 |  | 0.650 (0.489) | 0.183 |
| Owns Home | **-0.391***** (0.049) | 0.000 |  | 3.390*** (0.415) | 0.000 |
| Mother University Educated | **-0.296***** (0.048) | 0.000 |  | 5.977*** (0.377) | 0.000 |
| Maternal Psychological Distress | **0.080***** (0.005) | 0.000 |  | -0.141** (0.046) | 0.002 |
| Lives in London | **-0.166**** (0.063) | 0.008 |  | 1.338* (0.578) | 0.021 |
| Constant | **1.944***** (0.204) | 0.000 |  | 97.972*** (1.390) | 0.000 |
| **Random Effects** |  |  |  |  |  |
| **Level 2 (child)** |  |  |  |  |  |
| Between-child intercept variance | 1.129 (0.039) |  |  | 81.765 (2.897) |  |
| Between-child slope variance | 0.019 (0.001) |  |  | 1.280 (0.102) |  |
| Between-child intercept/slope variance covariance | -0.040 (0.006) |  |  | 0.949 (0.346) |  |
| **Level 3 (area)** |  |  |  |  |  |
| Variance | 0.017 (0.011) |  |  | 4.027 (0.692) |  |
| **Residual variance (level 1)** | 1.284 (0.031) |  |  | 90.093 (2.057) |  |
| **Note.** $\boldsymbol{b}$ **= unstandardised coefficients; SE = Standard Error. *p < .05. **p < .01. ***p < .001** | | | | | |

| ***Table S12***  *Fixed and Random Effects Estimates for Conduct Problems and Cognitive Ability (replacing urban/rural area with settlement type) (n = 6946)* | | | | | |
| --- | --- | --- | --- | --- | --- |
|  | Conduct Problems | |  | Cognitive Ability | |
|  | *b (SE)* | *p* |  | *b (SE)* | *p* |
| **Fixed Effects** |  |  |  |  |  |
| Age | **-0.228***** (0.007) | 0.000 |  | **0.477***** (0.109) | 0.000 |
| Age^2^ | **0.053***** (0.001) | 0.000 |  | **-** | - |
| Greenspace | 0.004 (0.011) | 0.739 |  | -0.127 (0.092) | 0.169 |
| Greenspace x Age | **0.003*** (0.001) | 0.015 |  | **-0.099***** (0.020) | 0.000 |
| England Disadvantaged (ref. England advantaged) | **0.214***** (0.044) | 0.000 |  | **-1.647**** (0.483) | 0.001 |
| England Ethnic (ref. England advantaged) | 0.041 (0.090) | 0645 |  | **-1.881*** (0.845) | 0.026 |
| Urban > 10k – less sparse (ref. Urban >10k – less sparse) | **1.297*** (0.515) | 0.012 |  | 4.261 (4.357) | 0.328 |
| Town and fringe – sparse (ref. Urban > 10k – less sparse) | -0.182 (0.157) | 0.247 |  | 0.073 (4.003) | 0.985 |
| Village, hamlet and isolated dwellings – sparse (ref. Urban > 10k – less sparse) | -0.278 (0.337) | 0.410 |  | 2.765 (1.491) | 0.064 |
| Town and fringe - less sparse (ref. Urban > 10k – less sparse) | 0.009 (0.065) | 0.890 |  | 0.940 (0.593) | 0.113 |
| Village, hamlet and isolated dwellings - less sparse (ref. Urban > 10k – less sparse) | -0.103 (0.066) | 0.115 |  | 0.897 (0.611) | 0.142 |
| Air Pollution (PM10) | -0.000 (0.011) | 0.997 |  | 0.019 (0.089) | 0.828 |
| Sole Access to a Garden | -0.091 (0.101) | 0.367 |  | 0.116 (0.541) | 0.831 |
| Female | **-0.331***** (0.032) | 0.000 |  | **1.934***** (0.271) | 0.000 |
| Ethnicity Mixed (ref. Ethnicity White) | -0.144 (0.085) | 0.089 |  | 0.083 (0.984) | 0.933 |
| Ethnicity Indian (ref. Ethnicity White) | -0.106 (0.110) | 0.335 |  | **-2.557*** (1.217) | 0.036 |
| Ethnicity Pakistani/Bangladeshi (ref. Ethnicity White) | -0.158 (0.081) | 0.050 |  | **-8.205***** (0.856) | 0.000 |
| Ethnicity Black (ref. Ethnicity White) | **-0.492**** (0.148) | 0.001 |  | **-4.331**** (1.249) | 0.001 |
| Ethnicity Other (ref. Ethnicity White) | **-0.502***** (0.116) | 0.000 |  | **-2.248*** (1.089) | 0.039 |
| Moved House | -0.029 (0.029) | 0.327 |  | -0.192 (0.294) | 0.515 |
| Below the poverty line | 0.199*** (0.054) | 0.000 |  | **-3.421***** (0.422) | 0.000 |
| Lives with both natural parents | -0.242*** (0.068) | 0.000 |  | 0.645 (0.492) | 0.190 |
| Owns Home | -0.387*** (0.048) | 0.000 |  | **3.369***** (0.492) | 0.000 |
| Mother University Educated | -0.302*** (0.048) | 0.000 |  | **5.974***** (0.416) | 0.000 |
| Maternal Psychological Distress | 0.080*** (0.005) | 0.000 |  | **-0.141**** (0.046) | 0.002 |
| Constant | 1.705*** (0.183) | 0.000 |  | **99.102***** (1.197) | 0.000 |
| **Random Effects** |  |  |  |  |  |
| **Level 2 (child)** |  |  |  |  |  |
| Between-child intercept variance | 1.127 (0.039) |  |  | 81.699 (2.910) |  |
| Between-child slope variance | 0.019 (0.001) |  |  | 1.279 (0.102) |  |
| Between-child intercept/slope variance covariance | -0.041 (0.006) |  |  | 0.947 (0.347) |  |
| **Level 3 (area)** |  |  |  |  |  |
| Variance | 0.020 (0.011) |  |  | 4.410 (0.742) |  |
| **Residual variance (level 1)** | 1.284 (0.031) |  |  | 90.076 (2.056) |  |
| **Note.** $\boldsymbol{b}$ **= unstandardised coefficients; SE = Standard Error. *p < .05. **p < .01. ***p < .001** | | | | | |


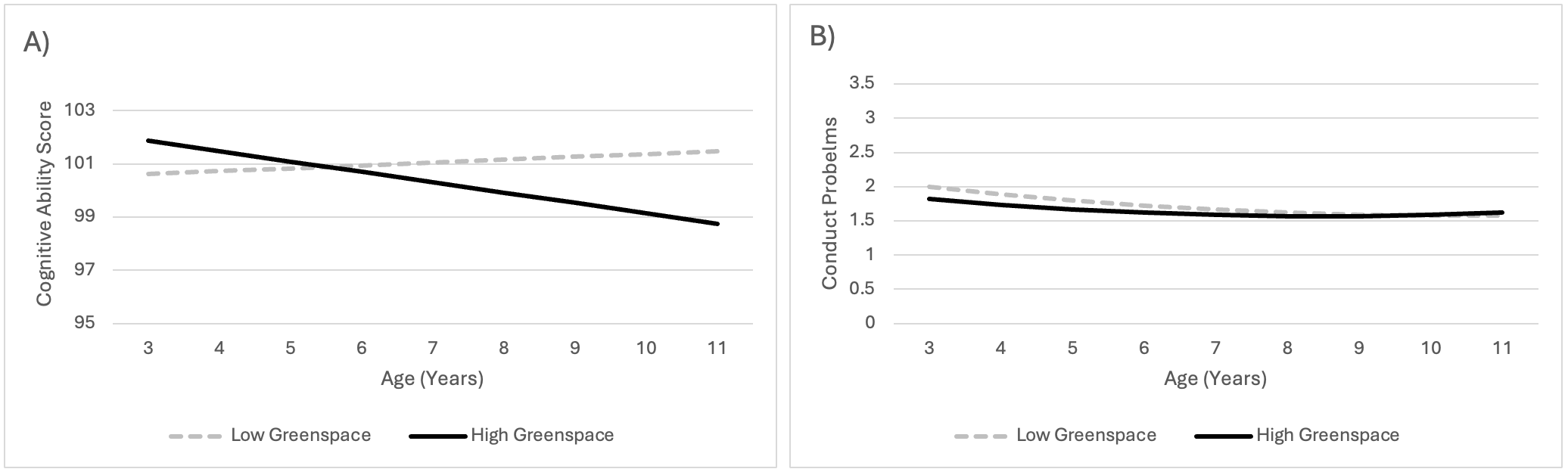


Figure S2. Predicted trajectories of cognitive ability (A) and conduct problems (B) by greenspace decile from ages 3 to 11 years old with the addition school type (independent or state), using the growth curve models fixed effects. The grey dashed line shows predicted scores in the lowest decile of greenspace, and the black solid line shows predicted scores in the highest decile of greenspace. Reference groups are used for categorical variables and means are used for continuous variables.

Supplementary Information: STROBE Statement—Checklist of items that should be included in reports of ***cohort studies***

|  | Item No | Recommendation | Done (x) |
| --- | --- | --- | --- |
| **Title and abstract** | 1 | (*a*) Indicate the study’s design with a commonly used term in the title or the abstract | Consider title: included MCS name, measured quantity of greenspace and use of growth curves |
|  |  | (*b*) Provide in the abstract an informative and balanced summary of what was done and what was found | Similarly, here, we have included that we measured quantity of greenspace, used MCS and growth curve models |
| Introduction | | |  |
| Background/rationale | 2 | Explain the scientific background and rationale for the investigation being reported | x |
| Objectives | 3 | State specific objectives, including any prespecified hypotheses | See present study section. |
| Methods | | |  |
| Study design | 4 | Present key elements of study design early in the paper | See title, abstract, present study section, and methods |
| Setting | 5 | Describe the setting, locations, and relevant dates, including periods of recruitment, exposure, follow-up, and data collection | See methods and supplementary information |
| Participants | 6 | (*a*) Give the eligibility criteria, and the sources and methods of selection of participants. Describe methods of follow-up | See methods and supplementary information |
|  |  | (*b*) For matched studies, give matching criteria and number of exposed and unexposed | NA |
| Variables | 7 | Clearly define all outcomes, exposures, predictors, potential confounders, and effect modifiers. Give diagnostic criteria, if applicable | See methods |
| Data sources/ measurement | 8* | For each variable of interest, give sources of data and details of methods of assessment (measurement). Describe comparability of assessment methods if there is more than one group | See methods |
| Bias | 9 | Describe any efforts to address potential sources of bias | See methods and results section “descriptive statistics” |
| Study size | 10 | Explain how the study size was arrived at | See methods and supplementary information |
| Quantitative variables | 11 | Explain how quantitative variables were handled in the analyses. If applicable, describe which groupings were chosen and why | See methods |
| Statistical methods | 12 | (*a*) Describe all statistical methods, including those used to control for confounding | See methods, specifically Statistical Approach and Table S1 |
|  |  | (*b*) Describe any methods used to examine subgroups and interactions | See methods, specifically Statistical Approach and Table S1 |
|  |  | (*c*) Explain how missing data were addressed | See methods, specifically Statistical Approach and readme file |
|  |  | (*d*) If applicable, explain how loss to follow-up was addressed | See methods, specifically Statistical Approach and readme file |
|  |  | (*e*) Describe any sensitivity analyses | See supplementary analysis and supplementary information |
| Results | | |  |
| Participants | 13* | (a) Report numbers of individuals at each stage of study—eg numbers potentially eligible, examined for eligibility, confirmed eligible, included in the study, completing follow-up, and analysed | See Tables 1 and 2 |
|  |  | (b) Give reasons for non-participation at each stage |  |
|  |  | (c) Consider use of a flow diagram | See Figure S1 |
| Descriptive data | 14* | (a) Give characteristics of study participants (eg demographic, clinical, social) and information on exposures and potential confounders | See Tables 1 and 2 |
|  |  | (b) Indicate number of participants with missing data for each variable of interest | See Tables 1 and 2, we have instead used number of participants with present data |
|  |  | (c) Summarise follow-up time (eg, average and total amount) | See Tables 1 and 2, we have average age instead which roughly translates to the follow-up times, |
| Outcome data | 15* | Report numbers of outcome events or summary measures over time | See Tables 1 and 2, we have reported mean and standard deviation or total number and percentage |
| Main results | 16 | (*a*) Give unadjusted estimates and, if applicable, confounder-adjusted estimates and their precision (eg, 95% confidence interval). Make clear which confounders were adjusted for and why they were included | See Tables 3, we used specific P values |
|  |  | (*b*) Report category boundaries when continuous variables were categorized | See Tables 1 and 2 |
|  |  | (*c*) If relevant, consider translating estimates of relative risk into absolute risk for a meaningful time period | NA |
| Other analyses | 17 | Report other analyses done—eg analyses of subgroups and interactions, and sensitivity analyses | See supplementary analysis and supplementary information |
| Discussion | | |  |
| Key results | 18 | Summarise key results with reference to study objectives | x |
| Limitations | 19 | Discuss limitations of the study, taking into account sources of potential bias or imprecision. Discuss both direction and magnitude of any potential bias | x |
| Interpretation | 20 | Give a cautious overall interpretation of results considering objectives, limitations, multiplicity of analyses, results from similar studies, and other relevant evidence | x |
| Generalisability | 21 | Discuss the generalisability (external validity) of the study results | x |
| Other information | | |  |
| Funding | 22 | Give the source of funding and the role of the funders for the present study and, if applicable, for the original study on which the present article is based | x |

*Give information separately for exposed and unexposed groups.

x refers to completion, but without further explanation, those with explanations show completion without the need for x.

**Note:** An Explanation and Elaboration article discusses each checklist item and gives methodological background and published examples of transparent reporting. The STROBE checklist is best used in conjunction with this article (freely available on the Web sites of PLoS Medicine at http://www.plosmedicine.org/, Annals of Internal Medicine at http://www.annals.org/, and Epidemiology at http://www.epidem.com/). Information on the STROBE Initiative is available at http://www.strobe-statement.org.
